# Arrhythmogenic cardiomyopathy as a myogenic disease: highlights from cardiomyocytes derived from human induced pluripotent stem cells

**DOI:** 10.3389/fphys.2023.1191965

**Published:** 2023-05-11

**Authors:** J. B. Reisqs, A. Moreau, Y. Sleiman, M. Boutjdir, S. Richard, P. Chevalier

**Affiliations:** ^1^ Cardiovascular Research Program, VA New York Harbor Healthcare System, Brooklyn, NY, United States; ^2^ Université de Montpellier, Institut National de la Santé et de la Recherche Médicale, Centre National de la Recherche Scientifique, PhyMedExp, Montpellier, France; ^3^ Department of Medicine, Cell Biology and Pharmacology, State University of New York Downstate Health Sciences University, NY, United States; ^4^ Department of Medicine, New York University School of Medicine, NY, United States; ^5^ Neuromyogene Institute, Claude Bernard University, Lyon 1, Villeurbanne, France; ^6^ Service de Rythmologie, Hospices Civils de Lyon, Lyon, France

**Keywords:** hiPSC-CM, arrhythmogenic cardiomyopathy, transdifferentiation, personalized medicine, electrophysiology

## Abstract

Arrhythmogenic cardiomyopathy (ACM) is an inherited cardiomyopathy characterized by the replacement of myocardium by fibro-fatty infiltration and cardiomyocyte loss. ACM predisposes to a high risk for ventricular arrhythmias. ACM has initially been defined as a desmosomal disease because most of the known variants causing the disease concern genes encoding desmosomal proteins. Studying this pathology is complex, in particular because human samples are rare and, when available, reflect the most advanced stages of the disease. Usual cellular and animal models cannot reproduce all the hallmarks of human pathology. In the last decade, human-induced pluripotent stem cells (hiPSC) have been proposed as an innovative human cellular model. The differentiation of hiPSCs into cardiomyocytes (hiPSC-CM) is now well-controlled and widely used in many laboratories. This hiPSC-CM model recapitulates critical features of the pathology and enables a cardiomyocyte-centered comprehensive approach to the disease and the screening of anti-arrhythmic drugs (AAD) prescribed sometimes empirically to the patient. In this regard, this model provides unique opportunities to explore and develop new therapeutic approaches. The use of hiPSC-CMs will undoubtedly help the development of precision medicine to better cure patients suffering from ACM. This review aims to summarize the recent advances allowing the use of hiPSCs in the ACM context.

## 1 Introduction

### 1.1 Clinical presentation

Arrhythmogenic Cardiomyopathy (ACM) is a rare genetic disease predisposing to a high risk for ventricular arrhythmias and heart failure (Basso et al., 2009; Bueno-Beti and Asimaki, 2021). Its prevalence is estimated between 1/1,000 and 1/5,000. The early clinical symptoms appear in young adults (Thiene et al., 1988; Roudijk et al., 2022). Historically, ACM has been described as affecting predominantly the right ventricle but this concept has evolved (Marcus et al., 1982). Recent studies showed that ACM can manifest early with a biventricular pattern or even as an isolated left ventricular dysfunction (Sen-Chowdhry et al., 2010; Pinamonti et al., 2014; Westphal et al., 2022). Four main disease stages are usually described. The first one is concealed with a risk of sudden death without structural abnormalities. During the second phase, structural changes appear gradually. The third and fourth phases are characterized by single and/or biventricular failure (Thiene et al., 2007; Asimaki et al., 2015).

The main feature of ACM is a loss of myocardium with fibro-fatty replacement. This phenomenon creates a conduction block causing asymmetric electrical conduction in the form of a loop through which the same electrical activity can propagate again and re-excite the tissue (Farré and Wellens, 2004). This process promotes electrical instability, thereby causing impaired ventricular mechanical function, potentially leading to sudden cardiac death (Corrado et al., 2017). The various forms of arrhythmias include palpitations, premature ventricular beat, bundle branch block, ventricular tachycardia, and ventricular fibrillation (Austin et al., 2019). On the ECG, ACM can manifest with a large QRS duration (>110 m), T-wave inversion, or also with the presence of an epsilon wave (Delmar and McKenna, 2010; Kalantarian et al., 2021). Structural heart remodeling manifests as fibrosis, fatty infiltration, and aneurysm, leading to ventricular dilation and decreased heart contraction (Kohela and van Rooij, 2022).

ACM is classified as an intercellular junction pathology (Basso et al., 2012; Marcus et al., 2013). In 50% of cases, ACM patients harbor a variant in genes coding for desmosomal proteins, including plakophilin-2 (*PKP2*, the most affected gene), desmoplakin (*DSP*), desmoglein-2 (*DSG2*), junction plakoglobin (*JUP*) and desmocollin-2 (*DSC2*). Alternatively, variants in genes coding for non-desmosomal proteins such as the ryanodine receptor 2 (*RYR2*), transforming growth factor reduced β-3 (*TGFβ3*), transmembrane protein 43 (*TMEM43*), desmin (*DES*), titin (*TTN*), phospholamban (*PLN*), lamin A/C (*LMNA*) and sodium channel (*SCN5A*) proteins have also been described (Quarta et al., 2011; Groeneweg et al., 2014; Lazzarini et al., 2015).

### 1.2 Desmosomes

Desmosomes are structures located at the intercalated discs in the myocardium and they are responsible for intercellular adhesion (Garrod and Chidgey, 2008; Nielsen et al., 2023). This desmosomal protein complex consequently ensures solid intercellular junctions and notably explains why these structures are mainly found in stretched tissues such as the skin or the heart (Holthöfer et al., 2007; Najor, 2018). Desmosomes were thought to have a specific physical role in intercellular adhesion, linking the intracellular cytoskeleton to the extracellular cadherins domain. The hypothesis regarding the pathophysiology of ACM implies the destabilization of the desmosome structure, due to variants in desmosomal protein, and may weaken the right myocardium stretch resistance (Delmar and McKenna, 2010). This process could lead to myocyte death and replacement with fibrofatty tissue due to the limited heart regeneration potential. Such a fibrofatty replacement, associated with inflammatory mechanisms, could provide an arrhythmogenic substrate (Smith et al., 2020). Several studies have used cells or animal models to decipher the mechanisms involved. However, the precise clinical and biological features of ACM remain to be elucidated. This review recapitulates the knowledge about ACM and the recent contributions of human-induced pluripotent stem cells (hiPSC) for both a better understanding of the disease and the comprehensive development of precision therapy.

## 2 Established molecular mechanisms in ACM

Various studies have focused on disturbances of intracellular pathways described for ACM related to desmosome destabilization (Austin et al., 2019). Desmosome alteration is one of the leading hypotheses of cardiomyocyte replacement in ACM. Indeed, the Wnt/β-catenin and Hippo pathways are altered because desmosomal variants can lead to fibro-fatty replacement (Chen et al., 2014). Studies also highlighted an increase in PPARγ expression in human cardiomyocytes from ACM patients (Djouadi et al., 2009). PPARγ gene is a known master regulator of adipogenesis (Rosen et al., 2002; Mal et al., 2021).

### 2.1 The Wnt pathway

**FIGURE 1:**
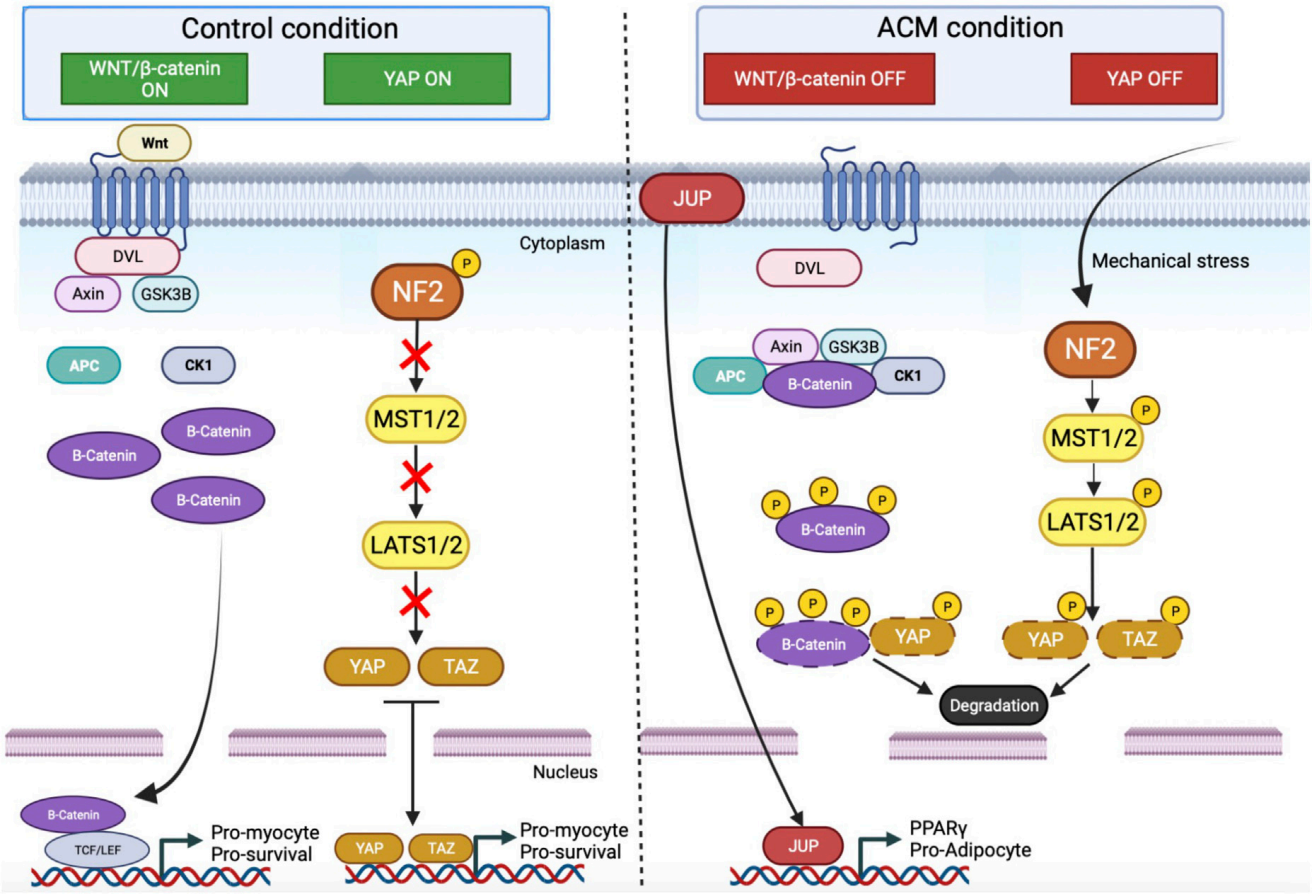
Wnt and Yap pathway and their interconnection between control and ACM condition. In control condition Wnt/β catenin and Yap pathway are ON. For Wnt signaling, the activation of the membrane receptor by Wnt ligands triggers the recruitment of Dishevelled (DVL). This complex also assembles cytoplasmic proteins, like Axin and Glycogen synthase 3β (GSK3β), and induces an accumulation of free β-catenin in the cytoplasm. β-catenin goes to the nucleus and binds with the transcription factors TCF/LEF to promote the expression of pro-myocyte genes. For the Yap pathway, NF2 is phosphorylated and inhibits the cascade of phosphorylation. YAP and TAZ are free into the cytoplasm and go to the nucleus to promote the expression of a pro-myocyte gene. In ACM conditions, these pathways are OFF. The β-catenin is sequestered in a molecular complex and hyperphosphorylated. Moreover, following mechanical stress, the Hippo pathway is activated which induces a cascade of phosphorylation up to YAP. Phosphorylated YAP will bind to the β-catenin. Both will be degraded by the proteasome. Finally, JUP, which is no longer retained on the membrane (due to desmosome destabilization), will be translocated into the nucleus, and enter into competition with β-catenin, triggering the expression of pro-adipogenic genes such as PPARγ. APC: Adenomatous polyposis coli; CK1: Casein kinase 1; DVL: Dishevelled; GSK3β: Glycogen synthase kinase 3β; LATS1/2: Large tumor suppressor kinase 1/2; MST1/2: Mammalian STE20-like protein kinase 1/2; PPARγ: Peroxisome proliferator-activated receptor gamma; TAZ: Transcriptional coactivator with PDZ-binding motif; TCF/LEF: T-cell factor/lymphoid enhancer factor family; YAP: Yes-associated protein.

The canonical Wnt signaling pathway regulates developmental processes during embryogenesis and is involved in the maintenance of adult tissue homeostasis (Logan and Nusse, 2004; Balatskyi et al., 2023). This signaling pathway is associated with cell differentiation, polarization, and migration during development (Steinhart and Angers, 2018). The canonical Wnt pathway plays also a pivotal role in adult cardiac remodeling by reactivation of the developmental program to maintain contractile function in the left ventricle (Bergmann, 2010). Canonical Wnt pathway activation inhibits the degradation of cytoplasmic β-catenin by the proteasome (Lustig and Behrens, 2003; DeBruine et al., 2017). The β-catenin can thus translocate into the nucleus and interact with the T-cell factor/Lymphoid-enhancer binding factor (Tcf/Lef) to activate the canonical Wnt signaling pathway (Figure 1) (Godsel et al., 2005; Nusse and Clevers, 2017). This pathway favors cell proliferation and regulates cell fate specification including cardiomyocyte differentiation (Rim et al., 2022).

In the ACM condition, an inhibition of the canonical Wnt pathway has been described in DSP-deficient mice and DSP-knockdown HL-1 cells leading to a morphological change of cardiomyocytes into adipocytes with lipid accumulation (Garcia-Gras et al., 2006). More recently, a study showed an alteration of canonical Wnt signaling in DSP-deficient zebrafish models (Giuliodori et al., 2018). Variants in genes coding for desmosomal proteins can induce a global desmosomal destabilization and lead to the cytoplasmic release of proteins usually retained at the plasma membrane. Using mice overexpressing the junction plakoglobin, Lombardi and coworkers further confirmed the ability of JUP to be translocated into the nucleus (Lombardi et al., 2011). The junction plakoglobin, also called γ-catenin competes with β-catenin which can also be found in both the cytoplasm and the nucleus. The nuclear re-localization of junctional plakoglobin prevents the interaction between β-catenin and Tcf/Lef and consequently affects the canonical Wnt pathway (Figure 1) (Lombardi et al., 2011). In cardiomyocytes, the suppression of Wnt signaling mainly promotes adipogenesis, thus potentially supporting lipid accumulation in the heart of ACM patients and the hypothesis of the transdifferentiation of cardiomyocytes into adipocytes. A relationship has been established in cellular models between the Wnt pathway and PPARγ expression (Liu et al., 2006; Parrotta et al., 2021). PPARγ is the master regulator for adipocyte differentiation, lipogenesis, and adipocyte survival (Cristancho et al., 2011; Lefterova et al., 2014). PPARγ is suspected to promote adipogenesis switch in ACM. In a study using DSP knockdown mice, Garcia-Gras et al. demonstrated a link between PPARγ overexpression and Wnt signaling suppression (Figure 1) (Garcia-Gras et al., 2006). This pathological mechanism may thus underlie the fibrofatty replacement characteristics of ACM.

### 2.2 The Hippo-Yap pathway

The Hippo-YAP pathway was discovered in 1995 using *Drosophila* genetic screening to isolate new genes involved in the regulation of cell proliferation, survival, and differentiation (Ma et al., 2019). This pathway involves a cascade of protein kinases. In the control condition, the kinases of Hippo are inactivated, YAP and TAZ are hypo-phosphorylated, then translocated into the nucleus to bind to DNA (Yu et al., 2015). Once YAP is in the nucleus, the YAP pathway is activated, promoting cell proliferation and resistance to apoptosis (Figure 1). However, when Hippo is activated by neurofibromin-2, which is a multifunctional protein involved in cell-cell and cell-matrix adhesions, mammalian STE20-like protein kinase 1 (MST1) and MST2 phosphorylate and activate the kinases large tumor suppressor homologs (LATS1 and LATS2). The MST and LATS are central kinases of the Hippo pathway. The LATS phosphorylates YAP and TAZ, inducing their degradation in the cytoplasm (Heallen et al., 2013). The YAP pathway is inactive, which inhibits gene expression (Figure 1).

ACM patients’ samples, mouse models, and PKP2 knockdown in HL-1 cells have demonstrated an aberrant activation of the Hippo signaling pathway leading to cytoplasmic retention of YAP (Chen et al., 2014). The cytoplasmic-retained YAP can interact with β-catenin and prevents its nuclear translocation into the nucleus (Figure 1). This further suppresses the Wnt signaling pathway leading to either the death of cardiomyocytes or their adipogenic trans-differentiation (Imajo et al., 2012; Hu and Pu, 2014). Despite its role, the Hippo-Yap pathway remains poorly studied, which warrants further investigations to elucidate the exact role of this pathway in ACM. This pathology is a pathology relying on cardiomyocytes connection and the activation/inhibition of Hippo-YAP is regulated by cell adhesion (Nishioka et al., 2009; Piquer-Gil et al., 2022).

### 2.3 Cell types involved in fibro-adipogenesis

Several hypotheses have emerged to explain the origin of fibro-adipose replacement in the ventricles. Early hypotheses focused on adult cardiac stem cells as a source (Stadiotti et al., 2017). Cardiac progenitor cells express desmosomal proteins. Studies in mice have shown the involvement of cells expressing the multipotent marker Isl-1 as a source of adipogenesis (Cohen et al., 2007; Lombardi et al., 2009). This hypothesis has been supported by the co-expression of Isl-1 markers and adipogenic transcription factors in the heart of ACM patients (Lombardi et al., 2009). The c-kit/Sca1 cellular progenitors have also been proposed as precursors of adipocytes (Lombardi et al., 2011). The c-kit and Sca1 markers are recognized markers of pluripotent stem cells in the hematopoietic system. Histological studies of transgenic mice over-expressing JUP showed an increase in the number of adipocytes and fibrosis in the heart (Lombardi et al., 2011). However, their low number suggests that only a small proportion of adipocytes originate from these cells (Sommariva et al., 2016).

Other studies have looked at the involvement of cardiac pluripotent cells such as cardiac mesenchymal stromal cells (Lombardi et al., 2016; Sommariva et al., 2016). These cells originate from the epicardium and participate in structural maintenance of the heart. They are pluripotent and involved in cardiac remodeling in pathological conditions. Their contribution to ACM has been demonstrated from studies of patient biopsies, from which these mesenchymal cells have been isolated and recultured. Under these conditions, these cells differentiated into adipocytes. (Lombardi et al., 2016). Another potential adipogenic source from pluripotent cells has been found in cardiac fibro-adipose progenitors. This previous study has shown that a mutation in a gene coding for a desmosomal protein can differentiate these cells into adipocytes. The authors estimated that 40% of adipocytes in the hearts of patients with AC originated from these progenitors (Lombardi et al., 2016).

**FIGURE 2:**
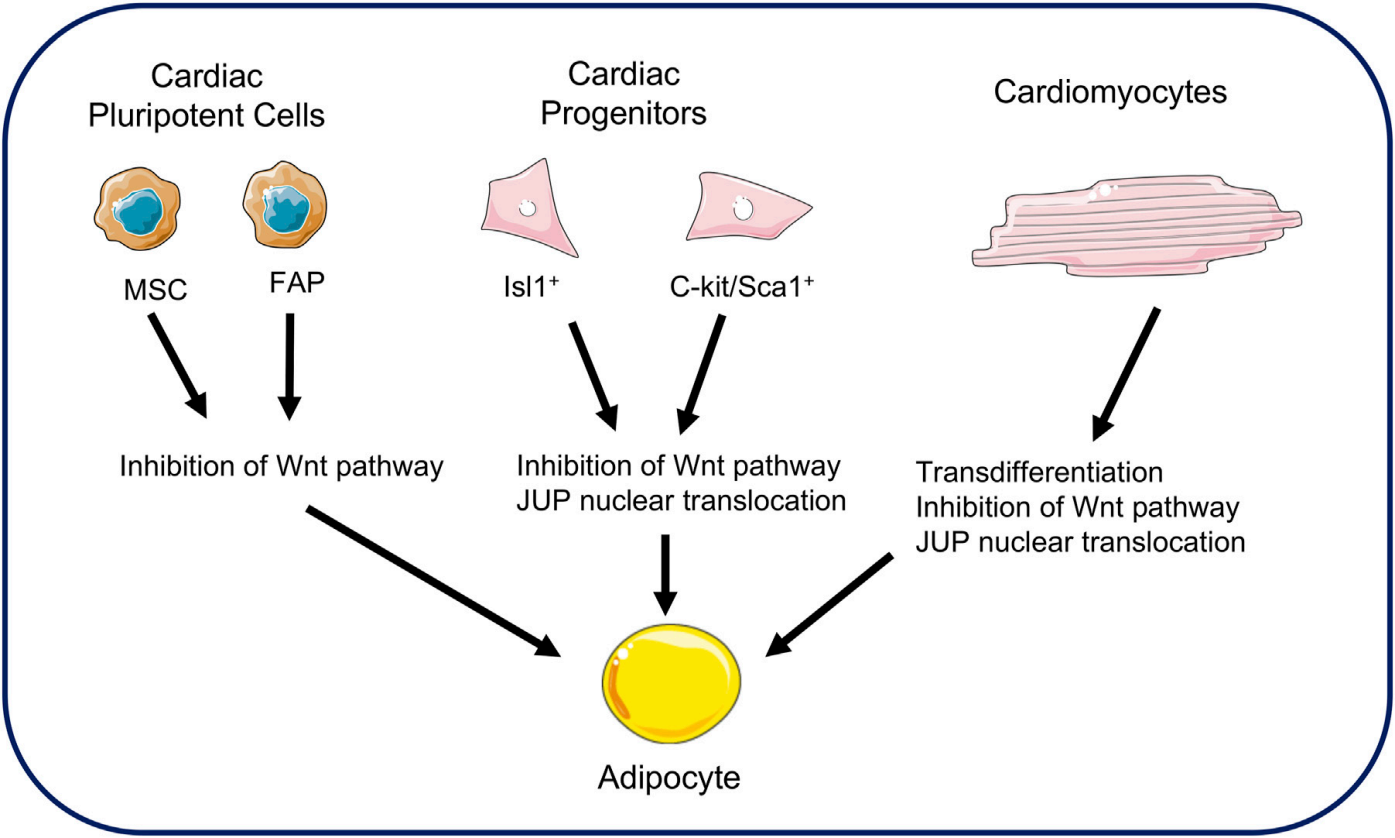
Cellular sources of adipocytes in arrhythmogenic cardiomyopathy. Fat replacement in ACM comes from several sources. Cardiac mesenchymal stromal cells and fibro-adipose progenitors may be sources of adipogenesis. Cardiac progenitors expressing Isl1+ and C-kit/Sca1+ are also sources of adipocytes. Finally, the transdifferentiation of cardiomyocytes into adipocytes is a possible hypothesis in fat replacement. All these sources demonstrate JUP involvement and Wnt pathway repression. (MSC: Mesenchymal stromal cells; FAP: Fibro-adipose progenitors).

Most studies of ACM mention the phenomenon of cardiomyocytes transdifferentiation as a source for fibro-adipose replacement (d’Amati et al., 2000; Fujita et al., 2008). D'Amati and collaborators were the first to report this phenomenon through histological, immunochemical, and ultrastructure analyzes in human cardiac samples (d’Amati et al., 2000). They reported positive labeling for vimentin, a protein expressed in adipocytes, in some cardiomyocytes. These cells would therefore be a transition cell type between cardiomyocytes and adipocytes. In addition, a second study reported this phenomenon of transdifferentiation where the myocardial cells had strong similarities with an adipocyte. Analysis of this group of cells reveals a polymorphic nuclear change, perinuclear vacuolation, and finally an accumulation of lipid droplets (Fujita et al., 2008). This phenomenon is supported by the idea that desmosome destabilization leads to the translocation of JUP from the membrane to the nucleus, thus inhibiting the action of β-catenin. The cellular source of fibro-adipose replacement in the ACM is shown in Figure 2.

### 2.4 Alterations of myocytes’ electrical activity as a critical pathogenic mechanism

Electrical remodeling, due to the destabilization of desmosomes, is a hallmark of ACM (Rizzo et al., 2012; Stevens et al., 2022). Desmosomes are physically close to gap junctions, belonging to the same macromolecular complex (Sato et al., 2011; Zhang J et al., 2021). Connexins are transmembrane proteins forming gap junctions enabling intercellular communication (Desplantez et al., 2007; Dhein and Salameh, 2021). In the diseased myocardium, alterations of gap junction organization and connexin expression are often observed (Severs et al., 2008; Chevalier et al., 2021). Studies in patients harboring a variant in *JUP* or *DSC2* highlighted abnormal connexin expression (Gehmlich et al., 2011). Similar findings have been observed *in-vitro* using RNA silencing technology to decrease the expression of PKP2 in neonatal rat ventricular myocytes. The loss of PKP2 expression leads to a drastic loss of Connexin43 (Oxford et al., 2007). Alterations in intercellular coupling via gap junctions are thus expected to settle a strong pro-arrhythmogenic substrate.

The desmosome/connexin macro-molecular complex also involves Na_v_1.5 voltage-gated sodium channels (Sato et al., 2009). In this study of neonatal rat ventricular myocytes, the knock-down of PKP2, using shRNA, revealed a loss of Na_v_1.5 and gap junctions, suggesting a link between desmosomes, gap junctions, and Na_v_1.5 at the intercalated disc. Variants in desmosomal proteins may also cause a drastic reduction in Na_v_1.5 sodium current (I_Na_) densities (Cerrone and Delmar, 2014). The use of a transgenic *DSG2* mouse model revealed that the reduction in I_Na_ density occurs before cardiomyocyte necrosis or fibrosis (Rizzo et al., 2012). Altogether, these results suggest that reduced I_Na_ densities due to variants in desmosomal proteins can establish an arrhythmogenic substrate and explain the conduction disturbances and arrhythmias seen early in ACM patients (Zaklyazminskaya and Dzemeshkevich, 2016). Moreover, I_Na_ reduction may be causal in the pathology rather than resulting from pathological phenotypic remodeling. This channel not only forms an ion pore but also plays a role in a functional adhesion/excitability complex with mechanical junctions. Depending on the protein interaction affected, a variant in the gene coding for Nav1.5 (SCN5A) may thereby cause a mixed electrical and structural phenotype in ACM (Te Riele et al., 2017).

The study of a cardiomyocyte-specific tamoxifen-induced PKP2 knockout mouse model demonstrated the reduced expression of genes controlling intracellular calcium, such as the Ryanodine Receptor 2 (*RYR2*) and the voltage-gated calcium channel (*CACNA1C*) gene (Cerrone et al., 2017). Variants in genes regulating calcium handling proteins were found in a cohort of patients diagnosed with ACM, particularly in genes encoding the RYR2 and phospholamban (PLN) (Tiso et al., 2001; van der Zwaag et al., 2012). Like for sodium channels, the loss of desmosomal genes provokes alterations in calcium handling, contributing to the development of arrhythmogenic events in ACM (Vallverdú-Prats et al., 2023). These findings indicate that cardiomyocytes not only undergo morphological and structural remodeling but also electrophysiological remodeling which contributes to the development of the pathology.

## 3 Highlights from hiPSC-CMs

Animal and cell models have considerably contributed to highlighting morphological and electrophysiological remodeling. However, these models do not always reproduce all features of human pathology because of different limitations. It is difficult to consider the successful development of new treatments using only those models. The technology developed by Pr. S. Yamanaka, allows the reprogramming of adult mouse or human fibroblasts into pluripotent stem cells using four transcription factors (*KLF4*, *OCT3/4*, *SOX2*, and *C-Myc*) (Takahashi et al., 2007; Yamanaka, 2012). The newly obtained pluripotent stem cells are named induced pluripotent stem cells (iPSC). The human iPSCs (hiPSC) express embryonic factors such as TRA1-60 or SSEA-1, maintain their pluripotency, and demonstrate high self-renewal capabilities. The method has rapidly been expanded and hiPSC can now be obtained from several original tissue types including blood or urine (Hou et al., 2013; Moreau et al., 2017; Shi et al., 2017).

The capacity to differentiate hiPSC into spontaneously beating cardiomyocytes was a major advance in the understanding of cardiac pathologies (Burridge et al., 2012). Indeed, hiPSC- derived cardiomyocytes (hiPSC-CM) are essential in the study of cardiomyopathies, channelopathies, and molecule screening during drug development or safety studies (Moretti et al., 2010; Sun et al., 2012; Moreau et al., 2017; Sleiman et al., 2020; Poulin et al., 2021; Ait Benichou et al., 2022; Jauvin et al., 2023).

At the early stage of culture (20 days), hiPSC-CMs exhibit immature morphological characteristics. Analysis by Transmission Electronic Microscopy (TEM) and immunofluorescence revealed a poorly organized contractile machinery, with a low number of myofibrils, which lacked alignment, immature Z-band, and T-tubule. However, with prolonged time in culture (360 days), cells become larger and more elongated, with increases in the density and alignment of myofibrils. Interestingly, the number of MLC2v-positive cells increases in the late stage of culture, indicating maturing of ventricular-type hiPSC-CM (Kamakura et al., 2013; Lundy et al., 2013). To go further, the use of molecules and new culture media makes it possible to obtain more mature cardiomyocytes (Feyen et al., 2020; Chirico et al., 2022). On this topic, targeting signaling pathways such as PPARα, Pitx2 or the metabolic switch from glucose to fatty acid makes it possible to work with cardiomyocytes presenting a more mature morphology and phenotype at an earlier stage. (Song et al., 2021; Bissoli et al., 2023).

The functional and electrophysiological features of hiPSC-CMs have been documented. Electrophysiological investigations revealed a heterogeneous population of cells characterized by nodal-, atrial- or ventricular-like action potentials (AP) (Zhang et al., 2009; Zhao et al., 2019). Several ionic currents have been described in iPSC-CMs, reflecting notably the presence and function of the major ionic channels underlying an action potential. These currents include the inward sodium and calcium (L- and T-type) currents, the transient outward potassium, and the rapid and slow delayed rectifier potassium currents (Honda et al., 2011; Zhao et al., 2018). In atrial and ventricular hiPSC-CMs, the expression of a hyperpolarization-activated cyclic nucleotide-gated (HCN) channel may contribute to spontaneous electrical activity. The density of the inward rectifier potassium current (I_K1_) was lower than in native human ventricular cardiomyocytes (Ma et al., 2011). Despite evidence for incomplete electrophysiological maturity, the hiPSC-CMs revealed the presence of a functional excitation-contraction coupling close to native cardiomyocytes (Itzhaki et al., 2011). The hiPSC-CMs, therefore, provide a great opportunity for studying cardiac pathology, due to their morphological and electrophysiological phenotypes close to native human cardiomyocytes and for drug screening (Doss and Sachinidis, 2019). The AP profile of hiPSC-CMs and consequently the activity of individual membrane currents during the AP, differs from that of native human cardiomyocytes, largely due to the almost negligible expression of I_K1_ (Hoekstra et al., 2012). To overcome this problem, an artificial method consists of artificially injecting the I_K1_ current into the cells to make them a more reliable model for investigating mechanisms underlying cardiac arrhythmias (Meijer van Putten et al., 2015). Improving the maturity of hiPSC-CM to be as close as possible to native cardiomyocytes remains a challenge that could be met in part thanks to the development of 3D culture approaches (Mannhardt et al., 2016). 3D bioprinting makes it possible to build organoids with a structure closer to native tissue. This technique has shown better cardiomyocyte morphology as well as improved electrophysiological function (Maiullari et al., 2018; Kupfer et al., 2020). Another method to develop a more mature model of hiPSC cardiomyocytes is the use of an extracellular matrix (ECM). Studies have shown that ECM improves electrical propagation velocity as well as action potential upstroke velocity, hiPSC-CM hypertrophy, and increased expression of SCN5A, Kir2.1, Cx43 and cardiac troponin I (Herron et al., 2016). The use of human ECM could encounter hiPSC-CM immaturity issues for optimal pre-clinical drug discoveries (Block et al., 2020). Finally, another study showed that the use of a cardioid platform allows a better understanding of the stages of cardiomyogenesis and improves the organization of hiPSC-CMs (Hofbauer et al., 2021).

**FIGURE 3:**
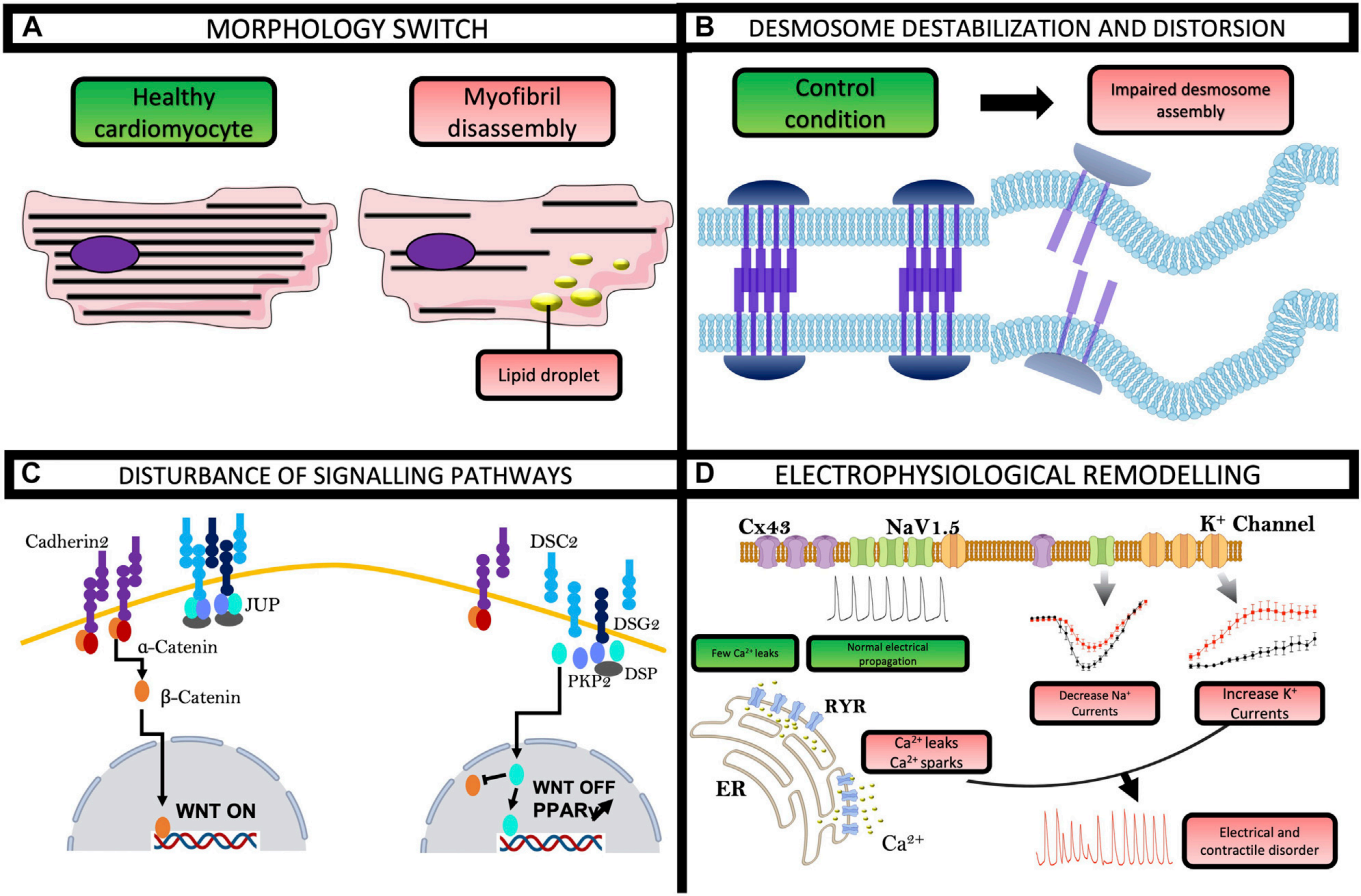
Highlights from hiPSC-CMs in ACM. These cells made it possible to recapitulate and highlight most of the hallmarks of the pathology, namely, **(A)** lipogenesis with a lipid droplet accumulation and **(B)** desmosome destabilization and distortion. **(C)** hiPSC-CMs also described a disturbance in Wnt and PPARγ signaling pathway. **(D)** In addition, hiPSC technology has shed light on direct electrophysiological disturbances that partly explain the occurrence of arrhythmias in ACM patients.

The hiPSCs model constitutes a revolutionizing method to obtain spontaneously contracting cardiomyocytes, which opens many avenues for studying cardiac pathologies. In 2013, Ma and co-authors were the first to differentiate hiPSC into cardiomyocytes using dermal fibroblasts from an ACM patient. Since, several studies using ACM-derived hiPSC-CMs showed that this cell type can recapitulate the key features of clinical pathology. Experiences by TEM and Oil Red O staining revealed clusters of lipid droplets in ACM hiPSC-CMs (Figure 3A) (Ma et al., 2013). TEM also showed a widened and distorted desmosome in ACM condition, which is one of the characteristics of the weakening desmosomal complex (Figure 3B) (Caspi et al., 2013). More specifically, using polymerase chain reaction (PCR) and immunostaining, these studies showed a significant decrease in the expression of PKP2, JUP, and connexin43. Kim and collaborators observed a nuclear localization of JUP and very low β-catenin activity, which has been previously described to induce adipogenic switch (Figure 3C) (Kim et al., 2013). Interestingly, these studies have shown an important role of PPARγ in the appearance of an ACM phenotype. The activation of this gene by rosiglitazone and indomethacin demonstrated an exaggerated lipogenesis and increased apoptosis in ACM hiPSC-CMs whereas the blockade of PPARγ rescued all ACM phenotypes (Wen et al., 2015). Besides morphological changes, other studies on hiPSC also revealed electrophysiological remodeling in ACM. The patch-clamp technique demonstrated a decrease in the amplitude and the maximal upstroke velocity of action potential in comparison with control hiPSC-CMs. These reductions involved a decrease in the peak I_Na_ in ACM hiPSC-CMs (Figure 3D) (El-Battrawy et al., 2018). Surprisingly, despite an increase in the rapid delayed rectifying potassium current (I_Kr_), there was no difference in the action potential duration (APD). This could also rely on hiPSC-CMs on their developmental stage or/and heterogeneity in their stage of differentiation (35 days) at the time of investigations. These ion channel problems were also found in another hiPSC line carrying a variant in the DSP gene (Gusev et al., 2020). The hiPSC-CMs from an ACM patient were also more sensitive to adrenergic stimulation than control cells. Isoprenaline shortened APD in ACM hiPSC-CM and epinephrine unleashed more arrhythmogenic events early after depolarization (EAD)-like or delayed after depolarization (DAD)-like (El-Battrawy et al., 2018). One publication demonstrated the link between I_Na_ and Wnt/B-catenin activity in ACM hiPSC-CMs with a variant in the gene encoding PKP2 (Khudiakov et al., 2020). To recapitulate, the PKP2 variant induced a significant reduction of Wnt activity and I_Na_ density, which was restored by the inhibition of Glycogen synthase kinase-3 beta (GSK3β) (Khudiakov et al., 2020). A new DSG2 variant in hiPSC-CM demonstrated an increased pro-inflammatory cytokine expression, accompanied by a shortened APD and a calcium transient decay reduced (Hawthorne et al., 2021). Collectively, the aberrant cytoskeletal organization, cytokine expression, electrophysiology, and calcium handling disturbance found in ACM-hiPSC-CMs could explain the arrhythmogenic mechanisms of the disease in ACM patients (Inoue et al., 2022).

hiPSCs are also used to explain therapies in patients. A study demonstrated the beneficial effect of the combination of sotalol and flecainide in ACM patients, using this cellular tool (Moreau et al., 2021). Indeed, the sotalol resulted in the normalization of APD by blocking potassium currents. Flecainide had the effect of decreasing cellular excitability by decreasing the number of aberrant calcium sparks. Moreover, this publication has shed light on an early repolarization disorder in some ACM patients (Chevalier et al., 2021; Moreau et al., 2021). Other studies use hiPSCs to demonstrate signaling pathway involvement in disease spread. A study shows the role of Nuclear Factor-κB (NF-κB) signaling in the inflammation of hiPSC harboring a variant in the *PKP2* gene (c.2013delC) (Chelko et al., 2019). This study defines inflammatory signaling which is activated in ACM and drives key features of the disease. Targeting inflammatory pathways may be an effective new mechanism-based therapy for ACM. Another study defines the role of PPARγ signaling in electrophysiological and calcium disturbances in ACM hiPSC-CMs and the inhibition of this pathway can rescue these troubles (Reisqs et al., 2022). A very recent publication demonstrates the beneficial effect of spironolactone, an anti-diuretic, to prevent the onset of arrhythmias (Reisqs et al., 2023). All the discoveries and advances obtained by hiPSCs are summarized in Table 1.

**TABLE 1:** Summary of studies carried out using hiPSC-CMs in ACM and associated discoveries.

Variant	Culture method	Findings	Highlights	Reference
PKP2 c.1841T>C	Embryoid Body (EB)	• Decreaseddesmosome gene expression	• ACM phenotype	Ma et al. (2013)
		• Lipid droplets accumulation	•Lipogenesis	
PKP2 c.972InsT/N	EB	• Reduced density in desmosomes	• ACM phenotype	Caspi et al. (2013)
		• Distorted desmosomes	• Lipogenesis	
		• Lipid droplets accumulation		
PKP2 c.2484C>T	EB	• JUP nuclear translocation	• Transdifferentiation	Kim et al. (2013)
		• Decreased β-catenin activity		
		• Increased PPARγ activity		
PKP2 c.2484C>T c.2013delC	EB	• Cardiomyocytes apoptosis	• Transdifferentiation	Wen et al. (2015)
		• Lipid droplets accumulation		
		• Co-activation of PPARγ and PPARα		
PKP2 c.2013delC	Monolayer	• hiPSC differentiation in epicardial cells	• Transdifferentiation	Kohela et al. (2021)
		• Spontaneous fibro-fatty cellular differentiation	• Epicardial cells differentiation into fibroblasts and fat cells	
DSG2 p.Gly638Arg	EB	• Lower upstroke in AP	• Electrophysiological remodeling	El-Battrawy et al. (2018)
		• Increase I_Kr_ current		
		• More sensitive to Isoprenaline		
DSP		• Lower amplitude of I_Na_ and I_CaL_ currents	• Electrophysiological remodeling	Gusev et al. (2020)
H1684R		• Higher I_to_ current		
		• Shortened AP		
PKP2 c.354delT		• Decreased Wnt/β catenin activity	• Electrophysiological remodeling	Khudiakov et al. (2020)
		• Decreased I_Na_ current	• Wnt/β catenin regulates I_Na_ current density	
PKP2 Truncating PKP2 mutation by CRISPR-Cas9	Monolayer	• Aberrant expression and localization of junctional components	• Sarcomeric disorganization	Zhang J et al. (2021)
		• Destabilization of sarcomere	• Electrophysiological remodeling	
		• Increased in APD	• Contractile defect	
DSG2 c.2358delA		• Increase in pro-inflammatory cytokine expression	• Inflammatory	Hawthorne et al. (2021)
		• Shortened AP	• Electrophysiological remodeling	
		• Shortened Ca^2+^ transients	• Ca^2+^ handling perturbation	
PKP2	Monolayer	• Impaired desmosome assembly	• Contractile defect	Inoue et al. (2022)
		• Disruption of Intercalated disc		
		• Decrease in the contractile function		
DSC2 c.394C>T	Monolayer	• Decrease of I_Na_ current	• Electrical instability	Moreau et al. (2021)
		• Increase of global I_K_ current	• AAD therapy (Sotalol and Flecainide)	
		• Shortened AP		
PKP2 c.2013delC	monolayer	• Pro-inflammation	• New potential signaling targeting NF-κB	Chelko et al. (2019)
		• Inhibition of NF-κB rescue		
DSC2 c.394C>T	Monolayer	• Electrophysiological and calcium abnormalities rescued by PPARγ inhibition (T0070907)	• New signaling pathway implications in electrophysiological and contractile defects	Reisqs et al. (2022)
DSC2 c.394C>T	Monolayer	• Electrophysiological and calcium abnormalities rescued by an anti-diuretic (Spironolactone)	• New potential therapeutic strategy by Spironolactone	Reisqs et al. (2023)
PLN R14del		• New hiPSC Generation		Vera et al. (2022)
DSP c.1386del		• New hiPSC Generation		Loiben et al. (2023)
DSP c.6687delA	EHT	• Structural and contractile defect	• 3D culture	Bliley et al. (2021)

## 4 Advantages and limitations of hiPSC-CM

The hiPSC-CMs system offers unique access to study cardiac cellular functions and signaling pathways directly related to a given patient with ACM, which is not possible to reproduce the complete genetic background of a given patient in any animal model. Features found in non-hiPSC-CM and hiPSC-CM models are summarized in Table 2. The hiPSC-CMs allow a more cell-centered approach and have brought to light the greater importance of the myogenic origin of arrhythmias. The hiPSC-CMs form a monolayer or an organoid which also enables a multicellular approach to contractile function (Feaster et al., 2022). Recent studies conducted on ACM-hiPSC-CM reproduced hallmarks of ACM, such as contractile apparatus defects accompanied by fibro-fatty and lipid accumulation. The electrical modifications of ACM-hiPSC-CM are likely to create conduction blocks facilitating reentry arrhythmias (Ten Tusscher and Panfilov, 2007). The hiPSC-CMs are therefore a suitable approach for comprehensive studies of ACM and use as a valuable model for therapeutic investigations. Moreover, a study reported that two patients diagnosed with ACM presented ventricular fibrillation without structural abnormalities (Blom et al., 2019). Interestingly, studies with ACM-hiPSC-CMs demonstrated electrical and calcium disturbances supporting the idea of direct involvement of cardiomyocytes in the genesis of arrhythmias and not only following structural and morphological remodeling (Kohela et al., 2021; Zhang K et al., 2021). Arrhythmias would result not only from fibro-fatty replacement disturbing electrical wave propagation in the heart but also directly from a profound modification of the cell’s electrical profile. Electrical disturbances would thus constitute a predominant mechanism to underly the early rhythm disturbances in ACM (Moreau et al., 2021). These suggestions link to a clinical study where patients presented electrical disturbances in absence of structural modifications in the ventricle (Gomes et al., 2012).

**TABLE 2:** Summar of the difference between non-hiPSC-CM and hiPSC-CM cells.

	Non-hiPSC-CM	hiPSC-CM
Pathway	• Suppression of Wnt/β-catenin	• Suppression of Wnt/β-catenin
	• Dysregulation of HIPPO/YAP pathway	• Dysregulation of HIPPO/YAP pathway
	• Upregulation of PPARγ	• Upregulation of PPARγ
		• Implication of NF-κB
		• Pro-inflammatory implication
Morphology	• Desmosome Destabilization	• Desmosome Destabilization
	• Lipid Accumulation	• Lipid Accumulation
	• Apoptosis	• Apoptosis
		• Transdifferentiation
Electrophysiology	• Reduced Nav1.5 current	• Reduced Nav1.5 current
		• Shortened Action Potential Duration
	• Decrease of Cx43 gene expression	• Increase in global potassium currents
		• Decrease and defect contractile function
		• Calcium handling disturbance
Therapy		• Combination of Sotalol and Flecainide
		• Spironolactone

**FIGURE 4:**
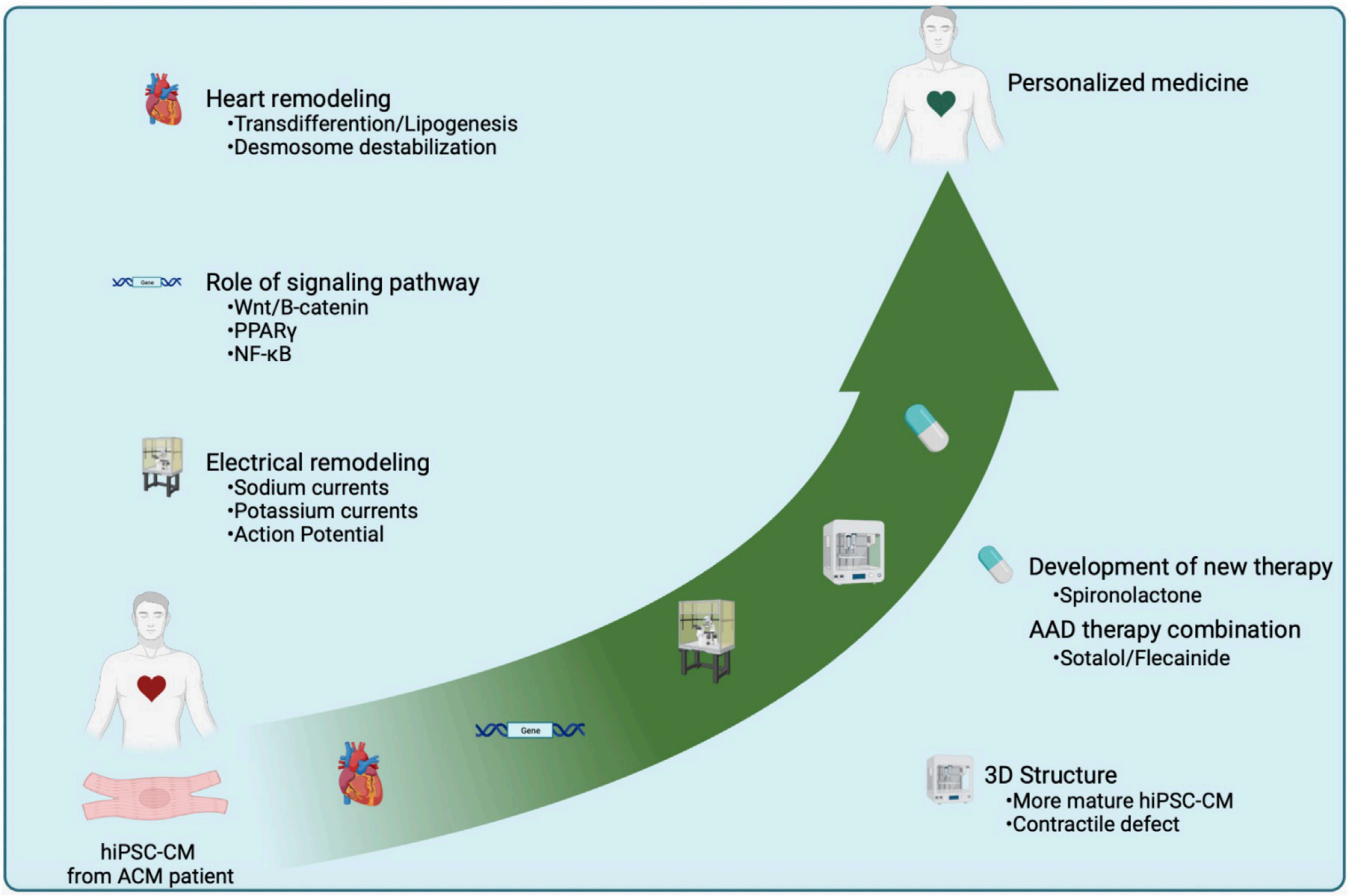
Central illustration summarizing the studies and the highlights obtained using hiPSC-CMs in the context of ACM. hiPSC-CM made it possible to summarize the hallmarks of pathology such as lipogenesis, electrical instability, and the discovery of the cardiomyocyte transdifferentiation phenomenon. This cellular model has allowed a better understanding of the use of AAD. A 3D culture approach refined the knowledge about the contractile defects in this pathology. The hiPSC-CMs have enabled a more cardiomyocyte-centric approach to gain more accurate information aiming at the development of personalized medicine. Finally, the discovery of new signaling pathways involved in ACM might reveal novel therapeutic approaches.

The hiPSC models open up immense prospects for progressing towards personalized medicine strategies for the diagnosis (functional and mechanistic at the cellular level) and the management of patients suffering from pathologies linked to variants. In ACM, one objective is to reduce the risk of arrhythmia for the patient. ICD placement and catheter ablation are frequently used in ACM patients. However, these methods do not prevent the occurrence of arrhythmias in these patients. That’s why we use the hiPSC model to find therapies to prevent the onset of ventricular arrhythmias. Antiarrhythmic drugs are therefore used to reduce this risk (Ermakov and Scheinman, 2015). However, studies on Anti-arrhythmic drugs (AAD) prescription for ACM patients are lacking, due to the rarity of cases and the complexity of this pathology limiting the information available to understand disease progression and improve the prevention and/or treatment of patients. In an ACM-hiPSC-CMs model, we demonstrated the potential beneficial effects of the combination of sotalol and flecainide, where the sotalol normalized APD while flecainide normalized calcium handling (Moreau et al., 2021). These results provided a mechanistic validation for the use of these drugs used sometimes empirically in patients with ACM. They also support the hiPSC-CMs system as a valuable tool to improve and rationalize patient treatments. Furthermore, the recapitulation and correction of this pathology using gene therapy in ACM-hiPSC-CM, support the development of personalized medicine and provide evidence for gene replacement therapy (Shiba et al., 2021). This cellular model can also be used to discover new therapeutic pathways like NF-κB or using the anti-diuretic (Figure 4) (Chelko et al., 2019; Reisqs et al., 2023).

Most studies were carried out based on PKP2 variants, which could limit the relevance on a larger scale and for other variants. However, the emergence of new hiPSC cell lines bearing other variants will allow us to refine our findings (Vera et al., 2022; Loiben et al., 2023). This could also help to elucidate a generalized pathological mechanism across all ACM genetic sources. In addition, these studies on hiPSC-CM were conducted between 30 and 60 days of culture. We have seen that a more prolonged culture (360 days) could improve cell maturity. However, we found the main highlights of the ACM at 30–60 days. In addition, we demonstrated a shortened APD in ACM-hiPSC-CM which shed light on the problem of QT duration in the ACM patient’s ECG (Moreau et al., 2021). The use of hiPSC-CM at 30–60 days is, therefore, suitable to translate the phenotype in patients. To overcome these problems of immaturity, the emergence of novel 3D culture models or culture conditions will improve cardiomyocytes’ phenotypes, and pathological modeling, and favor the development of new therapies (Bliley et al., 2021). This latter publication describes impaired contractility associated with a reduction in desmosome number in a 3D model made of hiPSC-CM carrying a *DSP* variant. Since hiPSC-CMs are cultured in a Petri dish, the involvement of other cells, such as c-kits, SMCs, or FAPs, in fibro-adipose replacement is difficult to assess. However, this approach has an essential advantage for studying the mechanisms in pure myocytes. These limits interpretations at the cardiomyocytes’ level but not in the context of tissue coherence integrating different cell types. These characteristics allowed the elucidation of potential transdifferentiation pathways in ACM-hiPSC-CM (Caspi et al., 2013; Ma et al., 2013; Reisqs et al., 2022). However, the co-culture of hiPSC-CM with cardiac fibroblasts from hiPSC could be useful to recreate a fibro-fatty infiltration in a dish to build a better model of ACM using hiPSC (Zhang et al., 2019).

## 5 Conclusion

This review summarizes the most advanced knowledge regarding the appropriate and relevant cellular model ACM pathological development. It further depicted several advances provided using hiPSC in studies of ACM. In addition to the fact that this model recapitulates the clinical hallmarks of the pathology, the results obtained using hiPSC, firstly, reinforce the previous cellular and animal models. Secondly, results obtained by hiPSC, are directly related to the patient. This model allows us to describe the pathology of the patient with the aim of more personalized medicine. This is of particular importance for cardiac diseases in general. Furthermore, hiPSC-CM is a cell-centered approach and deciphers the critical role of cardiomyocytes in the early manifestation and progression of ACM and serves as a unique patient-specific model for drug screening and hence therapy.

## Figures and Tables

**Table:** 

**AAD**	Anti-arrhythmic drugs
**ACM**	Arrhythmogenic Cardiomyopathy
**APD**	Action potential duration
**CACNA1C**	Voltage-gated calcium channel
**DAD**	Delayed after depolarization
**DES**	Desmin
**DSC2**	Desmocollin-2
**DSG2**	Desmoglein-2
**DSP**	Desmoplakin
**EAD**	Early after depolarization
**GSK3β**	Glycogen synthase kinase-3 beta
**hiPSC-CM**	human-induced pluripotent stem cells derived cardiomyocyte
**I**_**K1**_	Inward rectifier potassium current
**I**_**Kr**_	Rapid delayed rectifying potassium current
**I**_**Na**_	Sodium current
**JUP**	Plakoglobin
**LMNA**	Lamin A/C
**NF-κB**	Nuclear Factor-κB
**PCR**	Polymerase chain reaction
**PKP2**	Plakophilin-2
**PLN**	Phospholamban
**RYR2**	Ryanodine receptor 2
**SCN5A**	Sodium channel
**TCF/LEF**	T-cell factor/Lymphoid-enhancer binding factor
**TGFβ3**	Transforming growth factor reduced β-3
**TMEM43**	Transmembrane protein 43
**TTN**	Titin

## References

[B1] Ait Benichou S. Jauvin D. De Serres-Bérard T. Bennett F. Rigo F. Gourdon G. (2022). Enhanced delivery of ligand-conjugated antisense oligonucleotides (C16-HA-ASO) targeting dystrophia myotonica protein kinase transcripts for the treatment of myotonic dystrophy type 1. Hum. Gene Ther. 33, 810–820. 10.1089/hum.2022.069 35794764

[B2] Asimaki A. Kleber A. G. Saffitz J. E. (2015). Pathogenesis of arrhythmogenic cardiomyopathy. Can. J. Cardiol. 31, 1313–1324. 10.1016/j.cjca.2015.04.012 26199027 PMC4619183

[B3] Austin K. M. Trembley M. A. Chandler S. F. Sanders S. P. Saffitz J. E. Abrams D. J. (2019). Molecular mechanisms of arrhythmogenic cardiomyopathy. Nat. Rev. Cardiol. 16, 519–537. 10.1038/s41569-019-0200-7 31028357 PMC6871180

[B4] Balatskyi V. V. Sowka A. Dobrzyn P. Piven O. O. (2023). WNT/β-catenin pathway is a key regulator of cardiac function and energetic metabolism. Acta Physiol. oxf. Engl. 237, e13912. 10.1111/apha.13912 36599355

[B5] Basso C. Corrado D. Bauce B. Thiene G. (2012). Arrhythmogenic right ventricular cardiomyopathy. Circ. Arrhythm. Electrophysiol. 5, 1233–1246. 10.1161/CIRCEP.111.962035 23022706

[B6] Basso C. Corrado D. Marcus F. I. Nava A. Thiene G. (2009). Arrhythmogenic right ventricular cardiomyopathy. Lancet lond. Engl. 373, 1289–1300. 10.1016/S0140-6736(09)60256-7 19362677

[B7] Bergmann M. W. (2010). WNT signaling in adult cardiac hypertrophy and remodeling: Lessons learned from cardiac development. Circ. Res. 107, 1198–1208. 10.1161/CIRCRESAHA.110.223768 21071717

[B8] Bissoli I. D’Adamo S. Pignatti C. Agnetti G. Flamigni F. Cetrullo S. (2023). Induced pluripotent stem cell-based models: Are we ready for that heart in a dish? Front. Cell Dev. Biol. 11, 1129263. 10.3389/fcell.2023.1129263 36743420 PMC9892938

[B9] Bliley J. M. Vermeer M. C. S. C. Duffy R. M. Batalov I. Kramer D. Tashman J. W. (2021). Dynamic loading of human engineered heart tissue enhances contractile function and drives a desmosome-linked disease phenotype. Sci. Transl. Med. 13, eabd1817. 10.1126/scitranslmed.abd1817 34290054

[B10] Block T. Creech J. da Rocha A. M. Marinkovic M. Ponce-Balbuena D. Jiménez-Vázquez E. N. (2020). Human perinatal stem cell derived extracellular matrix enables rapid maturation of hiPSC-CM structural and functional phenotypes. Sci. Rep. 10, 19071. 10.1038/s41598-020-76052-y 33149250 PMC7643060

[B11] Blom L. J. Te Riele A. S. J. M. Vink A. Hauer R. N. W. Hassink R. J. (2019). Late evolution of arrhythmogenic cardiomyopathy in patients with initial presentation as idiopathic ventricular fibrillation. Hear. Case Rep. 5, 25–30. 10.1016/j.hrcr.2018.10.003 PMC6342730 30693201

[B12] Bueno-Beti C. Asimaki A. (2021). Histopathological features and protein markers of arrhythmogenic cardiomyopathy. Front. Cardiovasc. Med. 8, 746321. 10.3389/fcvm.2021.746321 34950711 PMC8688541

[B13] Burridge P. W. Keller G. Gold J. D. Wu J. C. (2012). Production of de novo cardiomyocytes: Human pluripotent stem cell differentiation and direct reprogramming. Cell Stem Cell 10, 16–28. 10.1016/j.stem.2011.12.013 22226352 PMC3255078

[B14] Caspi O. Huber I. Gepstein A. Arbel G. Maizels L. Boulos M. (2013). Modeling of arrhythmogenic right ventricular cardiomyopathy with human induced pluripotent stem cells. Circ. Cardiovasc. Genet. 6, 557–568. 10.1161/CIRCGENETICS.113.000188 24200905

[B15] Cerrone M. Delmar M. (2014). Desmosomes and the sodium channel complex: Implications for arrhythmogenic cardiomyopathy and brugada syndrome. Trends cardiovasc. Med. 24, 184–190. 10.1016/j.tcm.2014.02.001 24656989 PMC4099253

[B16] Cerrone M. Montnach J. Lin X. Zhao Y.-T. Zhang M. Agullo-Pascual E. (2017). Plakophilin-2 is required for transcription of genes that control calcium cycling and cardiac rhythm. Nat. Commun. 8, 106. 10.1038/s41467-017-00127-0 28740174 PMC5524637

[B17] Chelko S. P. Asimaki A. Lowenthal J. Bueno-Beti C. Bedja D. Scalco A. (2019). Therapeutic modulation of the immune response in arrhythmogenic cardiomyopathy. Circulation 140, 1491–1505. 10.1161/CIRCULATIONAHA.119.040676 31533459 PMC6817418

[B18] Chen S. N. Gurha P. Lombardi R. Ruggiero A. Willerson J. T. Marian A. J. (2014). The hippo pathway is activated and is a causal mechanism for adipogenesis in arrhythmogenic cardiomyopathy. Circ. Res. 114, 454–468. 10.1161/CIRCRESAHA.114.302810 24276085 PMC3946717

[B19] Chevalier P. Moreau A. Richard S. Janin A. Millat G. Bessière F. (2021). Short QT interval as a harbinger of an arrhythmogenic cardiomyopathy. Hear. Case Rep. 7, 734–738. 10.1016/j.hrcr.2021.07.010 PMC8602084 34820269

[B20] Chirico N. Kessler E. L. Maas R. G. C. Fang J. Qin J. Dokter I. (2022). Small molecule-mediated rapid maturation of human induced pluripotent stem cell-derived cardiomyocytes. Stem Cell Res. Ther. 13, 531. 10.1186/s13287-022-03209-z 36575473 PMC9795728

[B21] Cohen E. D. Wang Z. Lepore J. J. Lu M. M. Taketo M. M. Epstein D. J. (2007). Wnt/beta-catenin signaling promotes expansion of Isl-1-positive cardiac progenitor cells through regulation of FGF signaling. J. Clin. Invest. 117, 1794–1804. 10.1172/JCI31731 17607356 PMC1891000

[B22] Corrado D. Basso C. Judge D. P. (2017). Arrhythmogenic cardiomyopathy. Circ. Res. 121, 784–802. 10.1161/CIRCRESAHA.117.309345 28912183

[B23] Cristancho A. G. Lazar M. A. (2011). Forming functional fat: A growing understanding of adipocyte differentiation. Nat. Rev. Mol. Cell Biol. 12, 722–734. 10.1038/nrm3198 21952300 PMC7171550

[B24] d’Amati G. di Gioia C. R. Giordano C. Gallo P. (2000). Myocyte transdifferentiation: A possible pathogenetic mechanism for arrhythmogenic right ventricular cardiomyopathy. Arch. Pathol. Lab. Med. 124, 287–290. 10.5858/2000-124-0287-MT 10656741

[B25] DeBruine Z. J. Xu H. E. Melcher K. (2017). Assembly and architecture of the Wnt/β-catenin signalosome at the membrane. Br. J. Pharmacol. 174, 4564–4574. 10.1111/bph.14048 28941231 PMC5727340

[B26] Delmar M. McKenna W. J. (2010). The cardiac desmosome and arrhythmogenic cardiomyopathies: From gene to disease. Circ. Res. 107, 700–714. 10.1161/CIRCRESAHA.110.223412 20847325

[B27] Desplantez T. Dupont E. Severs N. J. Weingart R. (2007). Gap junction channels and cardiac impulse propagation. J. Membr. Biol. 218, 13–28. 10.1007/s00232-007-9046-8 17661127

[B28] Dhein S. Salameh A. (2021). Remodeling of cardiac gap junctional cell–cell coupling. Cells 10, 2422. 10.3390/cells10092422 34572071 PMC8465208

[B29] Djouadi F. Lecarpentier G. Hébert J.-L. Charron P. Bastin J. Coirault C. (2009). A potential link between peroxisome proliferator-activated receptor signalling and the pathogenesis of arrhythmogenic right ventricular cardiomyopathy. Cardiovasc. Res. 84, 83–90. 10.1093/cvr/cvp183 19497962

[B30] Doss M. X. Sachinidis A. (2019). Current challenges of iPSC-based disease modeling and therapeutic implications. Cells 8, 403. 10.3390/cells8050403 31052294 PMC6562607

[B31] El-Battrawy I. Zhao Z. Lan H. Cyganek L. Tombers C. Li X. (2018). Electrical dysfunctions in human-induced pluripotent stem cell-derived cardiomyocytes from a patient with an arrhythmogenic right ventricular cardiomyopathy. Europace. 20, f46–f56. 10.1093/europace/euy042 29566126

[B32] Ermakov S. Scheinman M. (2015). Arrhythmogenic right ventricular cardiomyopathy - antiarrhythmic therapy. Arrhythmia Electrophysiol. Rev. 4, 86–89. 10.15420/aer.2015.04.02.86 PMC4711546 26835106

[B33] Farré J. Wellens H. J. (2004). Philippe coumel: A founding father of modern arrhythmology. Europace 6, 464–465. 10.1016/j.eupc.2004.06.001 15384205

[B34] Feaster T. K. Casciola M. Narkar A. Blinova K. (2022). Evaluation of cardiac contractility modulation therapy in 2D human stem cell-derived cardiomyocytes. J. Vis. Exp. JoVE. 190, 64848. 10.3791/64848 36591970

[B35] Feyen D. A. M. McKeithan W. L. Bruyneel A. A. N. Spiering S. Hörmann L. Ulmer B. (2020). Metabolic maturation media improve physiological function of human iPSC-derived cardiomyocytes. Cell Rep. 32, 107925. 10.1016/j.celrep.2020.107925 32697997 PMC7437654

[B36] Fujita S. Terasaki F. Otsuka K. Katashima T. Kanzaki Y. Kawamura K. (2008). Markedly increased intracellular lipid droplets and disruption of intercellular junctions in biopsied myocardium from a patient with arrhythmogenic right ventricular cardiomyopathy. Heart Vessels 23, 440–444. 10.1007/s00380-008-1079-0 19037594

[B37] Garcia-Gras E. Lombardi R. Giocondo M. J. Willerson J. T. Schneider M. D. Khoury D. S. (2006). Suppression of canonical Wnt/beta-catenin signaling by nuclear plakoglobin recapitulates phenotype of arrhythmogenic right ventricular cardiomyopathy. J. Clin. Invest. 116, 2012–2021. 10.1172/JCI27751 16823493 PMC1483165

[B38] Garrod D. Chidgey M. (2008). Desmosome structure, composition and function. Biochim. Biophys. Acta 1778, 572–587. 10.1016/j.bbamem.2007.07.014 17854763

[B39] Gehmlich K. Syrris P. Peskett E. Evans A. Ehler E. Asimaki A. (2011). Mechanistic insights into arrhythmogenic right ventricular cardiomyopathy caused by desmocollin-2 mutations. Cardiovasc. Res. 90, 77–87. 10.1093/cvr/cvq353 21062920 PMC3058729

[B40] Giuliodori A. Beffagna G. Marchetto G. Fornetto C. Vanzi F. Toppo S. (2018). Loss of cardiac Wnt/β-catenin signalling in desmoplakin-deficient AC8 zebrafish models is rescuable by genetic and pharmacological intervention. Cardiovasc. Res. 114, 1082–1097. 10.1093/cvr/cvy057 29522173

[B41] Godsel L. M. Hsieh S. N. Amargo E. V. Bass A. E. Pascoe-McGillicuddy L. T. Huen A. C. (2005). Desmoplakin assembly dynamics in four dimensions: Multiple phases differentially regulated by intermediate filaments and actin. J. Cell Biol. 171, 1045–1059. 10.1083/jcb.200510038 16365169 PMC2171300

[B42] Gomes J. Finlay M. Ahmed A. K. Ciaccio E. J. Asimaki A. Saffitz J. E. (2012). Electrophysiological abnormalities precede overt structural changes in arrhythmogenic right ventricular cardiomyopathy due to mutations in desmoplakin-A combined murine and human study. Eur. Heart J. 33, 1942–1953. 10.1093/eurheartj/ehr472 22240500 PMC3409421

[B43] Groeneweg J. A. van der Heijden J. F. Dooijes D. van Veen T. a. B. van Tintelen J. P. Hauer R. N. (2014). Arrhythmogenic cardiomyopathy: Diagnosis, genetic background, and risk management. Neth. Heart J. Mon. J. Neth. Soc. Cardiol. Neth. Heart Found. 22, 316–325. 10.1007/s12471-014-0563-7 PMC4099433 24817548

[B44] Gusev K. Khudiakov A. Zaytseva A. Perepelina K. Makeenok S. Kaznacheyeva E. (2020). Impact of the DSP-H1684R genetic variant on ion channels activity in iPSC-derived cardiomyocytes. Cell. Physiol. biochem. Int. J. Exp. Cell. Physiol. biochem. Pharmacol. 54, 696–706. 10.33594/000000249 32706220

[B45] Hawthorne R. N. Blazeski A. Lowenthal J. Kannan S. Teuben R. DiSilvestre D. (2021). Altered electrical, biomolecular, and immunologic phenotypes in a novel patient-derived stem cell model of desmoglein-2 mutant ARVC. J. Clin. Med. 10, 3061. 10.3390/jcm10143061 34300226 PMC8306340

[B46] Heallen T. Morikawa Y. Leach J. Tao G. Willerson J. T. Johnson R. L. (2013). Hippo signaling impedes adult heart regeneration. Dev. Camb. Engl. 140, 4683–4690. 10.1242/dev.102798 PMC3833428 24255096

[B47] Herron T. J. Rocha A. M. D. Campbell K. F. Ponce-Balbuena D. Willis B. C. Guerrero-Serna G. (2016). Extracellular matrix-mediated maturation of human pluripotent stem cell-derived cardiac monolayer structure and electrophysiological function. Circ. Arrhythm. Electrophysiol. 9, e003638. 10.1161/CIRCEP.113.003638 27069088 PMC4833010

[B48] Hoekstra M. Mummery C. L. Wilde A. A. M. Bezzina C. R. Verkerk A. O. (2012). Induced pluripotent stem cell derived cardiomyocytes as models for cardiac arrhythmias. Front. Physiol. 3, 346. 10.3389/fphys.2012.00346 23015789 PMC3449331

[B49] Hofbauer P. Jahnel S. M. Papai N. Giesshammer M. Deyett A. Schmidt C. (2021). Cardioids reveal self-organizing principles of human cardiogenesis. Cell 184, 3299–3317. 10.1016/j.cell.2021.04.034 34019794

[B50] Holthöfer B. Windoffer R. Troyanovsky S. Leube R. E. (2007). Structure and function of desmosomes. Int. Rev. Cytol. 264, 65–163. 10.1016/S0074-7696(07)64003-0 17964922

[B51] Honda M. Kiyokawa J. Tabo M. Inoue T. (2011). Electrophysiological characterization of cardiomyocytes derived from human induced pluripotent stem cells. J. Pharmacol. Sci. 117, 149–159. 10.1254/jphs.11038fp 22027094

[B52] Hou P. Li Y. Zhang X. Liu C. Guan J. Li H. (2013). Pluripotent stem cells induced from mouse somatic cells by small-molecule compounds. Science 341, 651–654. 10.1126/science.1239278 23868920

[B53] Hu Y. Pu W. T. (2014). Hippo activation in arrhythmogenic cardiomyopathy. Circ. Res. 114, 402–405. 10.1161/CIRCRESAHA.113.303114 24481838 PMC4034381

[B54] Imajo M. Miyatake K. Iimura A. Miyamoto A. Nishida E. (2012). A molecular mechanism that links Hippo signalling to the inhibition of Wnt/β-catenin signalling. EMBO J. 31, 1109–1122. 10.1038/emboj.2011.487 22234184 PMC3297994

[B55] Inoue H. Nakamura S. Higo S. Shiba M. Kohama Y. Kondo T. (2022). Modeling reduced contractility and impaired desmosome assembly due to plakophilin-2 deficiency using isogenic iPS cell-derived cardiomyocytes. Stem Cell Rep. 17, 337–351. 10.1016/j.stemcr.2021.12.016 PMC8828557 35063130

[B56] Itzhaki I. Rapoport S. Huber I. Mizrahi I. Zwi-Dantsis L. Arbel G. (2011). Calcium handling in human induced pluripotent stem cell derived cardiomyocytes. PloS One 6, e18037. 10.1371/journal.pone.0018037 21483779 PMC3069979

[B57] Jauvin D. Pierre M. Boutjdir M. Puymirat J. Chahine M. (2023). Generation of four myotonic dystrophy type 1 patient iPSC lines (CBRCULi002-A, CBRCULi003-A, CBRCULi004-A, CBRCULi005-A) and a control (CBRCULi001-A) derived from lymphoblastoids cell lines. Stem Cell Res. 67, 103037. 10.1016/j.scr.2023.103037 36739767

[B58] Kalantarian S. Åström Aneq M. Svetlichnaya J. Sharma S. Vittinghoff E. Klein L. (2021). Long-term electrocardiographic and echocardiographic progression of arrhythmogenic right ventricular cardiomyopathy and their correlation with ventricular tachyarrhythmias. Circ. Heart Fail. 14, e008121. 10.1161/CIRCHEARTFAILURE.120.008121 34550004

[B59] Kamakura T. Makiyama T. Sasaki K. Yoshida Y. Wuriyanghai Y. Chen J. (2013). Ultrastructural maturation of human-induced pluripotent stem cell-derived cardiomyocytes in a long-term culture. Circ. J. 77, 1307–1314. 10.1253/circj.CJ-12-0987 23400258

[B60] Khudiakov A. Zaytseva A. Perepelina K. Smolina N. Pervunina T. Vasichkina E. (2020). Sodium current abnormalities and deregulation of Wnt/β-catenin signaling in iPSC-derived cardiomyocytes generated from patient with arrhythmogenic cardiomyopathy harboring compound genetic variants in plakophilin 2 gene. Biochim. Biophys. Acta Mol. Basis Dis. 1866, 165915. 10.1016/j.bbadis.2020.165915 32768677

[B61] Kim C. Wong J. Wen J. Wang S. Wang C. Spiering S. (2013). Studying arrhythmogenic right ventricular dysplasia with patient-specific iPSCs. Nature 494, 105–110. 10.1038/nature11799 23354045 PMC3753229

[B62] Kohela A. van Kampen S. J. Moens T. Wehrens M. Molenaar B. Boogerd C. J. (2021). Epicardial differentiation drives fibro-fatty remodeling in arrhythmogenic cardiomyopathy. Sci. Transl. Med. 13, eabf2750. 10.1126/scitranslmed.abf2750 34550725

[B63] Kohela A. van Rooij E. (2022). Fibro-fatty remodelling in arrhythmogenic cardiomyopathy. Basic Res. Cardiol. 117, 22. 10.1007/s00395-022-00929-4 35441328 PMC9018639

[B64] Kupfer M. E. Lin W.-H. Ravikumar V. Qiu K. Wang L. Gao L. (2020). *In situ* expansion, differentiation, and electromechanical coupling of human cardiac muscle in a 3D bioprinted, chambered organoid. Circ. Res. 127, 207–224. 10.1161/CIRCRESAHA.119.316155 32228120 PMC8210857

[B65] Lazzarini E. Jongbloed J. D. H. Pilichou K. Thiene G. Basso C. Bikker H. (2015). The ARVD/C genetic variants database: 2014 update. Hum. Mutat. 36, 403–410. 10.1002/humu.22765 25676813

[B66] Lefterova M. I. Haakonsson A. K. Lazar M. A. Mandrup S. (2014). PPARγ and the global map of adipogenesis and beyond. Trends Endocrinol. Metab. Tem. 25, 293–302. 10.1016/j.tem.2014.04.001 24793638 PMC4104504

[B67] Liu J. Wang H. Zuo Y. Farmer S. R. (2006). Functional interaction between peroxisome proliferator-activated receptor gamma and beta-catenin. Mol. Cell. Biol. 26, 5827–5837. 10.1128/MCB.00441-06 16847334 PMC1592783

[B68] Logan C. Y. Nusse R. (2004). The Wnt signaling pathway in development and disease. Annu. Rev. Cell Dev. Biol. 20, 781–810. 10.1146/annurev.cellbio.20.010403.113126 15473860

[B69] Loiben A. Friedman C. E. Chien W.-M. Stempien-Otero A. Lin S. Yang K.-C. (2023). Generation of human iPSC line from an arrhythmogenic cardiomyopathy patient with a DSP protein-truncating variant. Stem Cell Res. 66, 102987. 10.1016/j.scr.2022.102987 36481506 PMC9900081

[B70] Lombardi R. Chen S. N. Ruggiero A. Gurha P. Czernuszewicz G. Z. Willerson J. T. (2016). Cardiac fibro-adipocyte progenitors express desmosome proteins and preferentially differentiate to adipocytes upon deletion of the desmoplakin gene. Circ. Res. 119, 41–54. 10.1161/CIRCRESAHA.115.308136 27121621 PMC4920717

[B71] Lombardi R. da Graca Cabreira-Hansen M. Bell A. Fromm R. R. Willerson J. T. Marian A. J. (2011). Nuclear plakoglobin is essential for differentiation of cardiac progenitor cells to adipocytes in arrhythmogenic right ventricular cardiomyopathy. Circ. Res. 109, 1342–1353. 10.1161/CIRCRESAHA.111.255075 22021931 PMC3237769

[B72] Lombardi R. Dong J. Rodriguez G. Bell A. Leung T. K. Schwartz R. J. (2009). Genetic fate mapping identifies second heart field progenitor cells as a source of adipocytes in arrhythmogenic right ventricular cardiomyopathy. Circ. Res. 104, 1076–1084. 10.1161/CIRCRESAHA.109.196899 19359597 PMC2767296

[B73] Lundy S. D. Zhu W.-Z. Regnier M. Laflamme M. A. (2013). Structural and functional maturation of cardiomyocytes derived from human pluripotent stem cells. Stem Cells Dev. 22, 1991–2002. 10.1089/scd.2012.0490 23461462 PMC3699903

[B74] Lustig B. Behrens J. (2003). The Wnt signaling pathway and its role in tumor development. J. Cancer Res. Clin. Oncol. 129, 199–221. 10.1007/s00432-003-0431-0 12707770 PMC12161963

[B75] Ma D. Wei H. Lu J. Ho S. Zhang G. Sun X. (2013). Generation of patient-specific induced pluripotent stem cell-derived cardiomyocytes as a cellular model of arrhythmogenic right ventricular cardiomyopathy. Eur. Heart J. 34, 1122–1133. 10.1093/eurheartj/ehs226 22798562

[B76] Ma J. Guo L. Fiene S. J. Anson B. D. Thomson J. A. Kamp T. J. (2011). High purity human-induced pluripotent stem cell-derived cardiomyocytes: Electrophysiological properties of action potentials and ionic currents. Am. J. Physiol. Heart Circ. Physiol. 301, H2006–H2017. 10.1152/ajpheart.00694.2011 21890694 PMC4116414

[B77] Ma S. Meng Z. Chen R. Guan K.-L. (2019). The hippo pathway: Biology and pathophysiology. Annu. Rev. Biochem. 88, 577–604. 10.1146/annurev-biochem-013118-111829 30566373

[B78] Maiullari F. Costantini M. Milan M. Pace V. Chirivì M. Maiullari S. (2018). A multi-cellular 3D bioprinting approach for vascularized heart tissue engineering based on HUVECs and iPSC-derived cardiomyocytes. Sci. Rep. 8, 13532. 10.1038/s41598-018-31848-x 30201959 PMC6131510

[B79] Mal S. Dwivedi A. R. Kumar V. Kumar N. Kumar B. Kumar V. (2021). Role of peroxisome proliferator-activated receptor gamma (PPARγ) in different disease States: Recent updates. Curr. Med. Chem. 28, 3193–3215. 10.2174/0929867327666200716113136 32674727

[B80] Mannhardt I. Breckwoldt K. Letuffe-Brenière D. Schaaf S. Schulz H. Neuber C. (2016). Human engineered heart tissue: Analysis of contractile force. Stem Cell Rep. 7, 29–42. 10.1016/j.stemcr.2016.04.011 PMC4944531 27211213

[B81] Marcus F. I. Edson S. Towbin J. A. (2013). Genetics of arrhythmogenic right ventricular cardiomyopathy: A practical guide for physicians. J. Am. Coll. Cardiol. 61, 1945–1948. 10.1016/j.jacc.2013.01.073 23500315

[B82] Marcus M. Gh F. G G. R F. Jl L. C M. (1982). Right ventricular dysplasia: A report of 24 adult cases. Circulation 65, 384–398. 10.1161/01.cir.65.2.384 7053899

[B83] Meijer van Putten R. M. E. Mengarelli I. Guan K. Zegers J. G. van Ginneken A. C. G. Verkerk A. O. (2015). Ion channelopathies in human induced pluripotent stem cell derived cardiomyocytes: A dynamic clamp study with virtual Ik1. Front. Physiol. 6, 7. 10.3389/fphys.2015.00007 25691870 PMC4315032

[B84] Moreau A. Boutjdir M. Chahine M. (2017). Induced pluripotent stem-cell-derived cardiomyocytes: Cardiac applications, opportunities, and challenges. Can. J. Physiol. Pharmacol. 95, 1108–1116. 10.1139/cjpp-2016-0726 28350968

[B85] Moreau A. Reisqs J.-B. Delanoe-Ayari H. Pierre M. Janin A. Deliniere A. (2021). Deciphering DSC2 arrhythmogenic cardiomyopathy electrical instability: From ion channels to ECG and tailored drug therapy. Clin. Transl. Med. 11, e319. 10.1002/ctm2.319 33784018 PMC7908047

[B86] Moretti A. Bellin M. Welling A. Jung C. B. Lam J. T. Bott-Flügel L. (2010). Patient-specific induced pluripotent stem-cell models for long-QT syndrome. N. Engl. J. Med. 363, 1397–1409. 10.1056/NEJMoa0908679 20660394

[B87] Najor N. A. (2018). Desmosomes in human disease. Annu. Rev. Pathol. Mech. Dis. 13, 51–70. 10.1146/annurev-pathol-020117-044030 29414250

[B88] Nielsen M. S. van Opbergen C. J. M. van Veen T. A. B. Delmar M. (2023). The intercalated disc: A unique organelle for electromechanical synchrony in cardiomyocytes. Physiol. Rev. 2023, 2022. 10.1152/physrev.00021.2022 PMC10191137 36731030

[B89] Nishioka N. Inoue K. Adachi K. Kiyonari H. Ota M. Ralston A. (2009). The Hippo signaling pathway components Lats and Yap pattern Tead4 activity to distinguish mouse trophectoderm from inner cell mass. Dev. Cell 16, 398–410. 10.1016/j.devcel.2009.02.003 19289085

[B90] Nusse R. Clevers H. (2017). Wnt/β-Catenin signaling, disease, and emerging therapeutic modalities. Cell 169, 985–999. 10.1016/j.cell.2017.05.016 28575679

[B91] Oxford E. M. Musa H. Maass K. Coombs W. Taffet S. M. Delmar M. (2007). Connexin43 remodeling caused by inhibition of plakophilin-2 expression in cardiac cells. Circ. Res. 101, 703–711. 10.1161/CIRCRESAHA.107.154252 17673670

[B92] Parrotta E. I. Procopio A. Scalise S. Esposito C. Nicoletta G. Santamaria G. (2021). Deciphering the role of Wnt and rho signaling pathway in iPSC-derived ARVC cardiomyocytes by in silico mathematical modeling. Int. J. Mol. Sci. 22, 2004. 10.3390/ijms22042004 33670616 PMC7923182

[B93] Pinamonti B. Brun F. Mestroni L. Sinagra G. (2014). Arrhythmogenic right ventricular cardiomyopathy: From genetics to diagnostic and therapeutic challenges. World J. Cardiol. 6, 1234–1244. 10.4330/wjc.v6.i12.1234 25548613 PMC4278158

[B94] Piquer-Gil M. Domenech-Dauder S. Sepúlveda-Gómez M. Machí-Camacho C. Braza-Boïls A. Zorio E. (2022). Non coding RNAs as regulators of wnt/β-catenin and hippo pathways in arrhythmogenic cardiomyopathy. Biomedicines 10, 2619. 10.3390/biomedicines10102619 36289882 PMC9599412

[B95] Poulin H. Mercier A. Djemai M. Pouliot V. Deschenes I. Boutjdir M. (2021). iPSC-derived cardiomyocytes from patients with myotonic dystrophy type 1 have abnormal ion channel functions and slower conduction velocities. Sci. Rep. 11, 2500. 10.1038/s41598-021-82007-8 33510259 PMC7844414

[B96] Quarta G. Muir A. Pantazis A. Syrris P. Gehmlich K. Garcia-Pavia P. (2011). Familial evaluation in arrhythmogenic right ventricular cardiomyopathy: Impact of genetics and revised task force criteria. Circulation 123, 2701–2709. 10.1161/CIRCULATIONAHA.110.976936 21606390

[B97] Reisqs J.-B. Moreau A. Charrabi A. Sleiman Y. Meli A. C. Millat G. (2022). The PPARγ pathway determines electrophysiological remodelling and arrhythmia risks in DSC2 arrhythmogenic cardiomyopathy. Clin. Transl. Med. 12, e748. 10.1002/ctm2.748 35297182 PMC8926899

[B98] Reisqs J.-B. Moreau A. Sleiman Y. Charrabi A. Delinière A. Bessière F. (2023). Spironolactone as a potential new treatment to prevent arrhythmias in arrhythmogenic cardiomyopathy cell model. J. Pers. Med. 13, 335. 10.3390/jpm13020335 36836569 PMC9960914

[B99] Rim E. Y. Clevers H. Nusse R. (2022). The Wnt pathway: From signaling mechanisms to synthetic modulators. Annu. Rev. Biochem. 91, 571–598. 10.1146/annurev-biochem-040320-103615 35303793

[B100] Rizzo S. Lodder E. M. Verkerk A. O. Wolswinkel R. Beekman L. Pilichou K. (2012). Intercalated disc abnormalities, reduced Na(+) current density, and conduction slowing in desmoglein-2 mutant mice prior to cardiomyopathic changes. Cardiovasc. Res. 95, 409–418. 10.1093/cvr/cvs219 22764152

[B101] Rosen E. D. Hsu C.-H. Wang X. Sakai S. Freeman M. W. Gonzalez F. J. (2002). C/EBPalpha induces adipogenesis through PPARgamma: A unified pathway. Genes Dev. 16, 22–26. 10.1101/gad.948702 11782441 PMC155311

[B102] Roudijk R. W. Verheul L. Bosman L. P. Bourfiss M. Breur J. M. P. J. Slieker M. G. (2022). Clinical characteristics and follow-up of pediatric-onset arrhythmogenic right ventricular cardiomyopathy. JACC Clin. Electrophysiol. 8, 306–318. 10.1016/j.jacep.2021.09.001 35331425

[B103] Sato P. Y. Coombs W. Lin X. Nekrasova O. Green K. J. Isom L. L. (2011). Interactions between ankyrin-G, Plakophilin-2, and Connexin43 at the cardiac intercalated disc. Circ. Res. 109, 193–201. 10.1161/CIRCRESAHA.111.247023 21617128 PMC3139453

[B104] Sato P. Y. Musa H. Coombs W. Guerrero-Serna G. Patiño G. A. Taffet S. M. (2009). Loss of plakophilin-2 expression leads to decreased sodium current and slower conduction velocity in cultured cardiac myocytes. Circ. Res. 105, 523–526. 10.1161/CIRCRESAHA.109.201418 19661460 PMC2742576

[B105] Sen-Chowdhry S. Morgan R. D. Chambers J. C. McKenna W. J. (2010). Arrhythmogenic cardiomyopathy: Etiology, diagnosis, and treatment. Annu. Rev. Med. 61, 233–253. 10.1146/annurev.med.052208.130419 20059337

[B106] Severs N. J. Bruce A. F. Dupont E. Rothery S. (2008). Remodelling of gap junctions and connexin expression in diseased myocardium. Cardiovasc. Res. 80, 9–19. 10.1093/cvr/cvn133 18519446 PMC2533424

[B107] Shi Y. Inoue H. Wu J. C. Yamanaka S. (2017). Induced pluripotent stem cell technology: A decade of progress. Nat. Rev. Drug Discov. 16, 115–130. 10.1038/nrd.2016.245 27980341 PMC6416143

[B108] Shiba M. Higo S. Kondo T. Li J. Liu L. Ikeda Y. (2021). Phenotypic recapitulation and correction of desmoglein-2-deficient cardiomyopathy using human-induced pluripotent stem cell-derived cardiomyocytes. Hum. Mol. Genet. 30, 1384–1397. 10.1093/hmg/ddab127 33949662 PMC8283207

[B109] Sleiman Y. Souidi M. Kumar R. Yang E. Jaffré F. Zhou T. (2020). Modeling polymorphic ventricular tachycardia at rest using patient-specific induced pluripotent stem cell-derived cardiomyocytes. EBioMedicine 60, 103024. 10.1016/j.ebiom.2020.103024 32980690 PMC7519379

[B110] Smith E. D. Lakdawala N. K. Papoutsidakis N. Aubert G. Mazzanti A. McCanta A. C. (2020). Desmoplakin cardiomyopathy, a fibrotic and inflammatory form of cardiomyopathy distinct from typical dilated or arrhythmogenic right ventricular cardiomyopathy. Circulation 141, 1872–1884. 10.1161/CIRCULATIONAHA.119.044934 32372669 PMC7286080

[B111] Sommariva E. Brambilla S. Carbucicchio C. Gambini E. Meraviglia V. Dello Russo A. (2016). Cardiac mesenchymal stromal cells are a source of adipocytes in arrhythmogenic cardiomyopathy. Eur. Heart J. 37, 1835–1846. 10.1093/eurheartj/ehv579 26590176 PMC4912024

[B112] Song M.-H. Choi S.-C. Noh J.-M. Joo H. J. Park C.-Y. Cha J.-J. (2021). LEFTY-PITX2 signaling pathway is critical for generation of mature and ventricular cardiac organoids in human pluripotent stem cell-derived cardiac mesoderm cells. Biomaterials 278, 121133. 10.1016/j.biomaterials.2021.121133 34571434

[B113] Stadiotti I. Catto V. Casella M. Tondo C. Pompilio G. Sommariva E. (2017). Arrhythmogenic cardiomyopathy: The guilty party in adipogenesis. J. Cardiovasc. Transl. Res. 10, 446–454. 10.1007/s12265-017-9767-8 28983804 PMC5722955

[B114] Steinhart Z. Angers S. (2018). Wnt signaling in development and tissue homeostasis. Development 145, dev146589. 10.1242/dev.146589 29884654

[B115] Stevens T. L. Manring H. R. Wallace M. J. Argall A. Dew T. Papaioannou P. (2022). Humanized dsp ACM mouse model displays stress-induced cardiac electrical and structural phenotypes. Cells 11, 3049. 10.3390/cells11193049 36231013 PMC9562631

[B116] Sun N. Yazawa M. Liu J. Han L. Sanchez-Freire V. Abilez O. J. (2012). Patient-specific induced pluripotent stem cells as a model for familial dilated cardiomyopathy. Sci. Transl. Med. 4, 130ra47. 10.1126/scitranslmed.3003552 PMC3657516 22517884

[B117] Takahashi K. Tanabe K. Ohnuki M. Narita M. Ichisaka T. Tomoda K. (2007). Induction of pluripotent stem cells from adult human fibroblasts by defined factors. Cell 131, 861–872. 10.1016/j.cell.2007.11.019 18035408

[B118] Te Riele A. S. J. M. Agullo-Pascual E. James C. A. Leo-Macias A. Cerrone M. Zhang M. (2017). Multilevel analyses of SCN5A mutations in arrhythmogenic right ventricular dysplasia/cardiomyopathy suggest non-canonical mechanisms for disease pathogenesis. Cardiovasc. Res. 113, 102–111. 10.1093/cvr/cvw234 28069705 PMC5220677

[B119] Ten Tusscher K. H. W. J. Panfilov A. V. (2007). Influence of diffuse fibrosis on wave propagation in human ventricular tissue. EP Eur. 9, vi38–vi45. 10.1093/europace/eum206 17959692

[B120] Thiene G. Corrado D. Basso C. (2007). Arrhythmogenic right ventricular cardiomyopathy/dysplasia. Orphanet J. Rare Dis. 2, 45. 10.1186/1750-1172-2-45 18001465 PMC2222049

[B121] Thiene G. Nava A. Corrado D. Rossi L. Pennelli N. (1988). Right ventricular cardiomyopathy and sudden death in young people. N. Engl. J. Med. 318, 129–133. 10.1056/NEJM198801213180301 3336399

[B122] Tiso N. Stephan D. A. Nava A. Bagattin A. Devaney J. M. Stanchi F. (2001). Identification of mutations in the cardiac ryanodine receptor gene in families affected with arrhythmogenic right ventricular cardiomyopathy type 2 (ARVD2). Hum. Mol. Genet. 10, 189–194. 10.1093/hmg/10.3.189 11159936

[B123] Vallverdú-Prats M. Carreras D. Pérez G. J. Campuzano O. Brugada R. Alcalde M. (2023). Alterations in calcium handling are a common feature in an arrhythmogenic cardiomyopathy cell model triggered by desmosome genes loss. Int. J. Mol. Sci. 24, 2109. 10.3390/ijms24032109 36768439 PMC9917020

[B124] van der Zwaag P. A. van Rijsingen I. A. W. Asimaki A. Jongbloed J. D. H. van Veldhuisen D. J. Wiesfeld A. C. P. (2012). Phospholamban R14del mutation in patients diagnosed with dilated cardiomyopathy or arrhythmogenic right ventricular cardiomyopathy: Evidence supporting the concept of arrhythmogenic cardiomyopathy. Eur. J. Heart Fail. 14, 1199–1207. 10.1093/eurjhf/hfs119 22820313 PMC3475434

[B125] Vera C. D. Manhas A. Shenoy S. P. Wheeler M. T. Sallam K. Wu J. C. (2022). Generation of two induced pluripotent stem cell lines carrying the phospholamban R14del mutation for modeling ARVD/C. Stem Cell Res. 63, 102834. 10.1016/j.scr.2022.102834 35700631 PMC9476586

[B126] Wen J.-Y. Wei C.-Y. Shah K. Wong J. Wang C. Chen H.-S. V. (2015). Maturation-based model of arrhythmogenic right ventricular dysplasia using patient-specific induced pluripotent stem cells. Circ. J. Off. J. Jpn. Circ. Soc. 79, 1402–1408. 10.1253/circj.CJ-15-0363 25971409

[B127] Westphal D. S. Krafft H. Biller R. Klingel K. Gaa J. Mueller C. S. (2022). Myocarditis or inherited disease? - the multifaceted presentation of arrhythmogenic cardiomyopathy. Gene 827, 146470. 10.1016/j.gene.2022.146470 35381313

[B128] Yamanaka S. (2012). Induced pluripotent stem cells: Past, present, and future. Cell Stem Cell 10, 678–684. 10.1016/j.stem.2012.05.005 22704507

[B129] Yu F.-X. Zhao B. Guan K.-L. (2015). Hippo pathway in organ size control, tissue homeostasis, and cancer. Cell 163, 811–828. 10.1016/j.cell.2015.10.044 26544935 PMC4638384

[B130] Zaklyazminskaya E. Dzemeshkevich S. (2016). The role of mutations in the SCN5A gene in cardiomyopathies. Biochim. Biophys. Acta 1863, 1799–1805. 10.1016/j.bbamcr.2016.02.014 26916278

[B131] Zhang J. Liang Y. Bradford W. H. Sheikh F. (2021). Desmosomes: Emerging pathways and non-canonical functions in cardiac arrhythmias and disease. Biophys. Rev. 13, 697–706. 10.1007/s12551-021-00829-2 34765046 PMC8555023

[B132] Zhang J. Tao R. Campbell K. F. Carvalho J. L. Ruiz E. C. Kim G. C. (2019). Functional cardiac fibroblasts derived from human pluripotent stem cells via second heart field progenitors. Nat. Commun. 10, 2238. 10.1038/s41467-019-09831-5 31110246 PMC6527555

[B133] Zhang J. Wilson G. F. Soerens A. G. Koonce C. H. Yu J. Palecek S. P. (2009). Functional cardiomyocytes derived from human induced pluripotent stem cells. Circ. Res. 104, e30–e41. 10.1161/CIRCRESAHA.108.192237 19213953 PMC2741334

[B134] Zhang K K. Cloonan P. E. Sundaram S. Liu F. Das S. L. Ewoldt J. K. (2021). Plakophilin-2 truncating variants impair cardiac contractility by disrupting sarcomere stability and organization. Sci. Adv. 7, eabh3995. 10.1126/sciadv.abh3995 34652945 PMC8519574

[B135] Zhao Y. Rafatian N. Feric N. T. Cox B. J. Aschar-Sobbi R. Wang E. Y. (2019). A platform for generation of chamber-specific cardiac tissues and disease modeling. Cell 176, 913–927. 10.1016/j.cell.2018.11.042 30686581 PMC6456036

[B136] Zhao Z. Lan H. El-Battrawy I. Li X. Buljubasic F. Sattler K. (2018). Ion Channel expression and characterization in human induced pluripotent stem cell-derived cardiomyocytes. Stem Cells Int. 2018, 6067096–6067114. 10.1155/2018/6067096 29535773 PMC5835237

